# Publication bias, time-lag bias, and place-of-publication bias in social intervention research: An exploratory study of 527 Swedish articles published between 1990–2019

**DOI:** 10.1371/journal.pone.0281110

**Published:** 2023-02-06

**Authors:** Tina M. Olsson, Knut Sundell

**Affiliations:** 1 School of Health and Welfare, Jönköping University, Jönköping, Sweden; 2 Department of Social Work, Gothenburg University, Gothenburg, Sweden; 3 University of Gävle, Department of Social Work and Criminology, Gävle, Sweden; University of Sheffield, UNITED KINGDOM

## Abstract

Publication and related biases constitute serious threats to the validity of research synthesis. If research syntheses are based on a biased selection of the available research, there is an increased risk of producing misleading results. The purpose fo this study is to explore the extent of positive outcome bias, time-lag bias, and place-of-publication bias in published research on the effects of psychological, social, and behavioral interventions. The results are based on 527 Swedish outcome trials published in peer-reviewed journals between 1990 and 2019. We found no difference in the number of studies reporting significant compared to non-significant findings or in the number of studies reporting strong effect sizes in the published literature. We found no evidence of time-lag bias or place-of-publication bias in our results. The average reported effect size remained constant over time as did the proportion of studies reporting significant effects.

## Introduction

Social interventions are intentional change strategies designed to promote the healthy development or prevent the detrimental development of individuals, groups and society as a whole and may be developed and provided within one of several disciplines or contexts (e.g., social work, public health, psychology; [1]). Within the context of social intervention research, research syntheses (e.g., systematic reviews, meta-analyses) of randomized, controlled trials are considered the highest level of evidence and thus the strongest source of information regarding an intervention’s effectiveness [2]. Over the past decades there has been an exponential increase in the number of research synthesis articles published concerning social interventions (for example mental and beahvioral interventions; [3]), many of which are highly cited [e.g., 4–6]. This raises the issue of quality in research synthesis [e.g., 7] as the implications of poor quality could be significant due to the high value placed on these studies in informing policy and practice (e.g., the Cochrane Collaboration; Campbell Collaboration; the Swedish Agency for Health Technology Assessment and Assessment of Social Services, SBU; the National Institute for Health and Care Excellence, NICE, UK; the Agency for Healthcare Research and Quality, AHRQ, USA).

An important issue in research generally and within the context of research synthesis methodology specifically is the assessment and control of publication and related biases [e.g., time-lag bias, place of publication bias; 8–10] There is, however, heightened concern that the literature which lays the foundation for research synthesis is itself biased or incomplete [11] and that this bias is increasing [12]. Studies investigating change in publication bias over time have concluded that negative results are disappearing from the published literature across disciplines generally and within psychology, psychiatry, and biomedicine specifically [13–15]. The validity of research synthesis would be threatened if the studies upon which they are based represent a biased selection of all studies that have been conducted [16]. Recent investigation into publication bias in meta-analyses is, however, at odds with this view and have found little evidence of publication bias based on meta-meta-analysis of studies conducted in medicine and psychology [7, 17]. The purpose of this study is to increase our understanding of bias in the social intervention research literature using a body of efficacy and effectiveness research undertaken in Sweden and published between 1990 and 2019 as a case study.

Reasons for bias in the published literature include publication bias which occurs when research is undertaken but never published. Publication bias occurs when the direction or strength of a study’s findings influences the decision regarding whether to submit or accept manuscripts for publication [16]. This would result in, for example, a larger number of publications reporting significant results compared to non-significant results in the published literature [also known as positive-outcome bias; 13]. Similarly, time lag bias occurs when the speed of publication depends on the direction and strength of the trial results. For example, studies with significant results may be published earlier than those with non-significant results [e.g., 18]. Another related bias in the published literature is place of publication bias and is said to occur when the place of publication is associated with the direction or strength of the study findings [16]. When this bias is present, we would see research with significant and strong results being published in more prestigious journals and studies reporting null or weak findings being published in lower ranking journals. Regardless of the type of bias present, the results of publication and related biases render the published literature systematically different from the non-published literature [16]. Thus, threatening the validity of research syntheses based on this biased literature as unbiased estimates of intervention effectiveness are the cornerstone upon which research syntheses rest.

Within the broad field of healthcare, the issue of publication and related biases has been considered and investigated for several decades [e.g., 19, 20]. Here, studies have shown that the rate of publication is higher for studies reporting significant results versus studies reporting non-significant results. For example, in Ioannidis (1998) the rate of publication for studies reporting significant results (p ≤ 0.05) were between 60–98% compared to 20–85% for studies reporting non-significant results. Similarly, the pooled adjusted odds ratios for publication of studies reporting significant result has been found to be 2.54 (95% CI: 1.44–4.47) times higher than the odds of a study being published with non-significant results in one study [21] and 3.90 (95% CI: 2.68–5.68) times higher in another [22]. In the social sciences, recent attempts to estimate publication bias have been undertaken in economics [23], political science [24], sociology [25] and social science more broadly [26]. All of which suggest that the body of published research may be misleading due to publication bias, although specific attention to publication bias within social intervention research more broadly has not been previously addressed.

As a response to the growing understanding and empirical evidence supporting the issue of publication and related biases in research, several investigators have suggested that bias could be reduced through specific actions and reporting standards including: the prospective registration of trials, disclosure of conflicts and competing interests, publishing of supplementary materials, publishing of better powered studies, and enhanced research standards such as the use of CONSORT [27] in the reporting of trial results [e.g., 11, 16, 20, 28, 29]. Despite this work, little is known about the extent of publication, time lag, or place of publication biases in social science research generally [e.g., 7] or social intervention research specifically. The current study is exploratory and attempts to increase our understanding of publication, time lag, and place of publication bias and their relationship with the above suggested bias minimization efforts in the published social intervention research literature by investigating the following questions:

1. To what extent do we find evidence of positive outcome bias, time lag bias and place of publication bias in the published literature?
2. How has the proportion of significant vs non-significant study findings, time to publish and journal impact factor changed over time?
3. How has the reporting of study standards changed over time and what is the relationships between adherence to reporting standards and study findings?

## Materials and methods

### Search strategy

This study is a retrospective analysis and based on information provided in the published literature. In addition, it builds on a previous study investigating the methodological quality of Swedish effectiveness research published between 1990–2014 (see [30] for a detailed description of the search strategy used). All publications identified in the Sundell and Åhsberg [30] study are included in the current study. In addition, a search was made to identify studies published between 2015–2019 (additional details regarding the current study’s search strategy can be found in [31]).

First we contacted and conducted a bibliographic search of all 191 researchers that were previously identified as having published at least one effectiveness study during the period 1990–2014 [30]. Then we searched the six largest Swedish research funders: the Swedish Research Council for Health, Working Life and Welfare (Forte), the Swedish Research Council for Sustainable Development (Formas), the European Research Council (ERC), the Swedish Research Council (VR), the Swedish Innovation Agency (Vinnova), and the Swedish Crime Victim Authority (BRÅ), for grants awarded during the period 2015–2019 for effectiveness studies (search terms: evaluation, randomized). In addition, we conducted a search of planned effectiveness studies registered at clinicaltrials.com (search terms: Swed*, random*, effect*, evaluat*, RCT) as well as studies registered with ISRCTN at www.isrctn.com (search terms: Swed*, mental and behavioral disorder).

The researchers and studies identified in the search above were then included in searches in unified index EBSCO Discovery (Stockholm University library) (search terms: Swed*, random*, effect*, evaluat*, RCT). The studies identified in the search described in (30) and through the above described search [31] were then pooled and duplicates removed. If individual study results were reported in several publications, the first publication was used as the source for data extraction and coding. It should be noted that although our intent was to identify the population of studies published within the study period, we did not conduct a systematic review and therefore the population of studies contained here may be incomplete.

### Inclusion and exclusion criteria

#### Inclusion criteria

Publications were included in the current study if they had the following characteristics:

- The publication reported on a study which evaluated a behavioral, psychological, or social intervention regardless of the context within which it was delivered.
- The publication reported on a study which was undertaken in Sweden and the principal investigator was employed by a Swedish university or organization.
- The publication was from a scientific journal and was subjected to peer review prior to publication.
- The publication was published between 1990 and 2019.
- The study design reported in the publication was an efficacy, effectiveness, or field experiment using a randomized or non-randomized controlled design.

#### Exclusion criteria

Publications were excluded from the current study if they had any of the following characteristics:

- The study reported did not include an outcome measure at the client, patient, or user level (e.g., only included measures of professional behavior change).
- The study reported investigated an intervention designed to impact somatic health without including at least one behavioral, psychological, or social component.
- The study reported investigated and measured only pedagogical or didactical interventions (e.g., methods to teach children math skills).

### Adherence to reporting standards

We recorded data related to adherence to reporting standards as outlined in the CONSORT-statement [32], TREND-statement [33] and Prevention Science [34]. Items are reported individually and in a combined index consisting of 17 items. Coding of the articles was conducted independently by the authors of this study. The data extraction and coding instrument for this study can be found in S1 File. A subsample of 40 articles were independently coded by both authors and interrater reliability was investigated. Interrater reliability was assessed as ranging from substantial (Cohen’s *K* .58 - .79) to perfect (Cohen’s *K* .81–1.0). Additional details regarding interrater reliability can be found in S2 File.

### Data analysis

Data was analyzed with SAS version 9.4. Study characteristics are presented as frequencies. Changes over time and relationships between variables are investigated with *X*^2^ or ANOVA depending on variable characteristics. For analyses of changes over time, publications have been grouped by five-year period (e.g., 2000–2004, 2005–2009). Due to the low rate of publication during the 1990s, all articles published between 1990–1999 were grouped into one 10-year period. Effect sizes were calculated according to Lipsey and Wilson [35]. When not possible to extract an exact effect size estimate and the effect size were reported to be non-significant, the effect size was set to zero (n = 25). In seven trials the effect was reported to be significant, but the information reported was insufficient to calculate the effect size. In these cases, the effect was set to the average of all other studies (Cohen’s *d* = .48). Publication time was measured as the number of months between publication year and month and reported year and month of study start, funding or ethical approval. This information was missing in a fairly large number of studies (*n* = 188), especially in older studies. In the six cases the journal lacked an impact factor, it was set to zero in this analysis.

A total of 20 tests of significance on our primary outcomes were administered to investigate bias and changes in bias over time. To counteract the problem of multiple significance tests, all significance levels were adjusted according to Bonferroni’s correction. Therefore, significance is set at .003 in this study.

Since extreme effect sizes and publication time have a disproportionate influence on conclusions drawn from statistical analyses, we checked for outliers using Cook’s distance [36]. Three outliers were found on effect size and two on publication time. To reduce the impact of these outliers, their values were substituted by one that equaled the highest effect that fell within the normal range on Cohen’s d (2.3) and publication time (138 months). This had a negligible effect on study results.

As the sample of studies used in the analyses conducted here is heterogeneous, we chose not to use the funnel plot to assess publication bias. Instead, we explore the extent to which variation in study setting (social services, health services, psychiatry), prevention level (universal, selective, indicated) and study design (RCT, non-RCT) predict study findings using standard multiple regression and logistic regression depending on the dependent variable’s characteristics. Subgroups of studies found to significantly predict outcome are further explored with X^2^ or the standard independent samples t-test depending on data characteristics.

### Ethics statement

Ethics approval was not required nor sought for the current study.

## Results

### Study characteristics

We found 527 published effectiveness (456 randomized controlled trials, 71 non-randomized controlled trials) studies of behavioral, psychological, or social interventions. Table 1 presents a summary of the specific areas of practice these studies represent over time. Between 1990–1999 there were a handful of published articles. From 2000 the increase of published articles is steady.

**Table 1:** Published studies and associated field included in the analysis (n = 528).

Field	1990–1994 (n = 11)	1995–1999 (n = 20)	2000–2004 (n = 49)	2005–2009 (n = 96)	2010–2014 (n = 155)	2015–2019 (n = 196)	Total (n = 527)
Adult psychiatry	5	10	19	31	58	80	204
Somatic Healthcare	6	8	15	21	33	30	113
Social services/individual services	0	0	5	13	16	15	49
Social services/ family services	0	1	4	9	11	19	44
Child and youth psychiatry	0	0	2	7	8	20	37
Social services/elder services	0	1	3	7	13	9	33
Occupational theapy	0	0	0	6	7	11	24
Social services/disability services	0	0	0	0	2	6	8
Criminal justice	0	0	0	1	3	3	7
Dental care	0	0	1	1	1	1	4
Other	0	0	0	0	3	2	5
Total number of studies	11	20	50	96	155	196	528

The interventions evaluated targeted universal (14%), selected (13%) and indicated (73%) groups. Although some overlap in age groups, the main population was adults (73%), children and youth (19%) and elderly (7%). The most common comparison groups were other named interventions (40%), treatment-as-usual (23%) and wait-list or no service (37%). Included studies were published across 248 peer-reviewed journals with an average impact factor of 3.04 (SD 2.37; range 0.00–22.67).

### Positive outcome bias

Just over half (54%) of the included studies reported a significant effect of the assessed intervention versus a comparison group. Furthermore, one study found a significant effect in the comparison group’s favor.

### Time lag bias

The result on time between study start and publication is based on the 339 studies that reported a study start date (*n* = 160 non-significant findings; *n* = 179 significant findings). The publication time ranged from seven to 324 months, with an average publication time of 65.0 months (*SD* = 35.3). Time to publish did not change over time, *F*(4,338) = 2.03, *p* > .05.

There was no statistically significant difference in publication time between studies reporting significant (*M* = 60.8) vs. non-significant (*M* = 69.6) findings, *F*(1,338) = 5.32, *p* = .021. Controlling for length of follow-up (*M*_non-significnt_ = 14.2 months; *M*_significnt_ = 10.0 months) did not change this result, *F*(4, 335) = 0.72, *p* > 0.05. Study effect size was not statistically related to time to publish, *r*(*n* = 339) = -0.14, *p* = .010.

### Place of publication bias

There were no significant differences in impact factor between studies reporting significant effects and those that did not, *F* (1,526) = 6.72, *p* = .009. There were no significant differences in impact factor over time, *F* (4,526) = 1.95, *p* > .05.

### Study findings and effect size over time

The average effect size across studies was 0.48 (*SD* = 0.47). No differences were found in published effect size over time *F* (4,526) = 0.30, *p* > .05. There was no difference in reported significant effects over time, *x*^2^(4) = 3.67, *p* > .05.

### Adherence to reporting standards over time

Table 2 provides a summary of the extent to which included studies adhered to reporting standards over time. On average, studies reported 9.49 (*SD* 3.64) of the 17 standards assessed in this study. This number increased over time, *F (*4, 526) = 63.80; *p* < .001, from a low of 4.81 to a high of 11.61 reported standards in studies published between 1990–1999 compared to studies published between 2015–1019.

**Table 2:** Study report of individual and combined study standards over time.

Publication year	1990–99	2000–04	2005–09	2010–14	2015–19	*X*^2^ (4)
*N*	31	49	66	155	196	
*Study protocol and conflict of interest*						
Published study protocol (%)	0	6	4	30	58	128.72^b^
Conflict of interest statement (%)	3	4	20	53	83	187.99^b^
*Sample characteristics*						
Inclusion and exclusion criteria (%)	97	90	89	92	97	9.39
Demographic and clinical variables (%)	47	63	67	78	91	50.69^b^
Statistical power (%)	3	16	19	37	66	99.65^b^
*Data collection*						
Primary outcome stated (%)	6	18	27	53	72	105.51^b^
Psychometric properties of measures described (%)	28	41	54	58	79	51.13^b^
Blinded assessments (%)	19	35	31	45	39	10.99
Harmful effects measured (%)	6	12	5	13	19	12.65 ^a^
*Treatment fidelity*						
Implementation fidelity reported (%)	47	51	54	63	52	5.34
*Design*						
Controlled with randomization (%)	81	80	80	90	90	10.32 ^a^
Blinded assignment between research arms in randomized studies (%)	4	31	42	54	64	43.64^b^
Statistical control of selection bias in non-randomized studies (%)	-	-	30	-	40	5.82
*Analysis*						
Reported attrition (%)	44	59	70	84	93	66.35^b^
Intent-to-treat analysis (%)	6	35	49	63	63	48.92^b^
*Results*						
Descriptive statistics (%)	63	78	80	82	83	7.83
Effect size reported (%)	3	14	41	59	66	80.45^b^

^a^ p < 0.05

^b^ p < 0.001

### Relationship between adherence to reporting standards and study findings

Table 3 reports the relationship between study characteristics and significant vs. non-significant study findings. Of the 17 criteria assessed in this study, only seven reported effect size was significantly related to finding. Table 4 reports on the relationship between adherence to reporting standards and strength of result. Eight of the 17 criteria were significantly related to strength of study findings (Table 4).

**Table 3:** Study characteristics and published significant vs non-significant study findings (*n* = 522).

	Non-significant effect (%)	Significant effect (%)	chi^2^(1)	*p*
Published study protocol	30	32	0.27	n.s.
Conflict-of-interest statement	52	49	0.25	n.s.
Inclusion and exclusion criteria	93	93	0.00	n.s.
Demographic and clinical variables at baseline	77	79	0.23	n.s.
A priori power calculation	42	39	0.42	n.s.
Achieved study power^a^	57	61	0.32	n.s.
Reported a primary outcome variable	41	56	12.14	.001
Reported the psychometric properties of the measurement instruments used	51	70	19.82	.0001
Assessments were blinded	36	40	0.94	n.s.
Reported any harmful effects	9	17	7.66	.01
The intervention was implemented as intended	46	60	6.43	.05
The study was controlled with randomization	83	89	3.65	n.s.
Distribution of participants between study arms was blinded ^b^	45	56	5.17	.05
Attempts were made to minimize selection bias between study arms^c^	33	48	1.85	n.s.
Reported attrition	37	41	0.97	n.s.
Intent-to-treat analysis	49	59	4.68	n.s.
Descriptive statistics reported with results	76	84	6.09	.05
Effect size reported with results	38	61	29.66	.0001

^a^ *n* = 212

^b^ randomized trials only, *n* = 456

^c^ non-randomized trials only, *n* = 71

**Table 4:** Study characteristics and standardized mean differences (*n* = 527).

	Yes	No	*F*(1.526)	*p*
Published study protocol	.45	.49	0.72	n.s.
Conflict-of-interest statement	.42	.53	6.44	.05
Inclusion and exclusion criteria	.48	.43	0.37	n.s.
Demographic and clinical variables at baseline	.45	.58	7.31	.01
A priori power calculation	.42	.51	5.23	.05
Achieved study power^a^	.42	.41	0.08	n.s.
Reported a clear primary outcome variable	.53	.42	7.07	.01
Reported the psychometric properties of the measurement instruments used	.50	.43	3.10	n.s.
Assessments were blinded	.49	.47	0.35	n.s.
Reported any harmful effects	.70	.44	18.96	.0001
The intervention was implemented as intended	.52	.42	7.09	.01
The study was controlled with randomization	.50	.29	13.06	.001
Distribution of participants between study arms was blinded^b^	.50	.51	0.05	n.s.
Attempts were made to minimize selection bias between study arms^c^	.30	.28	0.06	n.s.
Reported attrition	.51	.45	1.73	n.s.
Intent-to-treat analysis	.49	.46	0.59	n.s.
Descriptive statistics reported with results	.52	.30	17.62	.0001
Effect size of primary outcome measure reported	.56	.39	17.73	.0001

^a^ *n* = 212

^b^ randomized trials only, *n* = 456

^c^ non-randomized trials only, *n* = 72

In addition, reporting standards were significantly related to all three indicators of publication bias. The number of reporting standards was higher in studies reporting significant effect sizes (*M* = 10,07) compared to those reporting non-significant effects (*M* = 8,81), *F*(1,526) = 16,04, *p* < .001. Time to publish was longer in studies reporting fewer reporting standards, *r*(*n* = 339) = -.34, *p* < .001, and journal impact factor was higher in studies reporting a higher number of reporting standards, *r*(*n* = 527) = 0.23, *p* < .001.

### Subgroup analyses

#### Positive outcome bias

Table 5 shows the results for the logistic regression used to assess the impact of setting, prevention level, and study design on the odds that included studies would report a significant or non-significant finding. The full model containing all predictors was statistically significant *X*^2^ (4, *n* = 527) = 18.66, *p* < 0.001, indicating that the model was able to distinguish between studies with and without significant effects. The model correctly classified 60% of cases. As shown in Table 5 only two of the independent variables made a unique statistically significant contribution to the model (health services, psychiatry). The strongest predictor of reporting a significant difference was whether the study was conducted within the psychiatry setting (*OR* = 1.82, *p* = .013). This indicated that the odds were 1.82 times greater that studies conducted within the psychiatry setting would report a significant finding, controlling for other factors in the model.

**Table 5:** Logistic regression model of the impact of setting, prevention level, and study design on the odds that included studies would report a significant or non-significant finding.

Model	B	Odds ratio	p
Health services	.585	1.796	.023
Psychiatry	.602	1.826	.013
Prevention level	.316	1.372	.159
Design (rct, non-rct)	.277	1.319	.306
Constant	-.757	.469	.006

No significant differences were found in the extent to which studies conducted within the health services setting reported significant vs non-significant findings *X*^2^ (1, *n* = 525) = 1.48, *p* > 0.05. A significant difference in studies reporting significant findings was found within the subgroup of psychiatric services setting X^2^ (1, *n* = 525) = 5.66, *p* = .017. In contrast, a significant difference in studies reporting non-significant findings was found with the subgroup of social services setting *X*^2^ (1, *n* = 525) = 15.19, *p* < .001

#### Place of publication

Table 6 shows the results from the standard multiple regression to assess the individual contribution of setting, prevention level, and study design on impact factor. Our model explains a statistically significant 2.4% of the variance in impact factor (*p* = .012). Study design (RCT, non-RCT) being the best predictor of variation in journal impact factor. There was a significant difference in journal impact factor for studies using an RCT (*M* = 3.17) vs non-RCT (*M* = 2.21) design *F*(1,526) = 10.14, *p* > 0.002.

**Table 6:** Standard multiple regression results of the individual contribution of setting, prevention level, and study design on impact factor.

Model	B	p
Constant	2.041	< .001
Health services	-0.085	.776
Psychiatry	0.151	.591
Prevention level	0.336	.199
Design (rct, non-rct)	0.818	.009

#### Time to publication

Table 7 shows the results for the standard multiple regression to assess the individual contribution of setting, prevention level, and study design on time to publication. Our model explains a statistically significant 7.3% of the variance in time to publication (*p* < .001). Study design (RCT, non-RCT) being the best predictor of variation in time to publication. There was a significant difference in time to publication for studies using an RCT (*M* = 60.8 months) vs non-RCT (*M* = 87.0 months) design *F*(1,338) = 27.00, *p* < 0.001.

**Table 7:** Standard multiple regression results of the individual contribution of setting, prevention level, and study design on time to publication.

Model	B	p
Constant	90.013	< .001
Health services	.939	.858
Psychiatry	-4.422	.358
Prevention level	-3.164	.484
Design (rct, non-rct)	-25.071	< .001

## Discussion

The purpose of this study was to explore the extent of publication bias (positive outcome bias) and related biases (time-lag bias and place-of-publication bias) in a cohort of studies published between 1990 and 2019. We aimed to answer three questions. First, we were interested in investigating the extent to which we find evidence of publication bias, time lag bias, and place of publication bias in the published literature. Publication bias has been investigated and found in primarily medical research. We found no differences in studies conducted within the health services between the number of studies reporting significant vs non-significant findings. A handful of prior studies have found publication bias within the social science literature [e.g., 23–26]. However, it is unclear how comparable these studies are to our investigation as they have investigated publication bias within economics, sociology, and social science more broadly. The study by Franco, et al. [26] included 249 individual studies across seven disciplines, the majority of which publish very little to no intervention research [see for example, 37]. Results showed that 20.5% of studies with null findings were published compared to 61.5% of studies with strong positive findings. Unfortunately, this study did not conduct any subgroup analyses by discipline, so it is difficult to make direct comparisons to our results.

We did not find a significant association between study findings and time to publish but did find that studies using a RCT design tend to be published faster than studies using a non-RCT design. Reviews of studies have found that on average the time-lag in publishing studies with null or negative results is approximately 2–3 years longer than studies reporting positive findings [38]. In our sample, the measured time to publish was non-significant over time. However, it should be remembered that there was a large attrition of studies due to lack of information regarding the start date of the studies included. That said, most of what we know about time lag bias is taken from the medical field. In their review of reviews, Hopewell et all [38] found seven reviews all investigating time lag bias. Although studies were eligible if they included any analysis of any aspect of the time to publication of clinical trials, no reviews were found within the social sciences. We were unable to find any study within the social sciences investigating the issue of time lag bias.

We found no significant association between study findings and place-of-publication but did find that studies using an RCT design tended to be published in journals with higher impact factors. This finding is difficult to interpret regarding the social science literature as comparative studies are lacking. Understanding the relationship between journal ranking and journal circulation and access is needed to better understand how place-of-publication within the social sciences might impact on publication bias more generally. It can be noted that systematic reviews do not exclude studies because of journal ranking, but instead on risk of bias, which repeatedly also occur in high-ranked journals. In our material, this is exemplified with a rather weak association between journal impact factor and adherence to reporting standards, and that some of the highest ranked journals included studies with quite low adherence to reporting standards as investigated in this study.

Second, we were interested in investigating the extent to which the report of significant vs. non-significant findings has changed over time. We found no difference in the proportion of studies reporting significant compared to non-significant results or in the proportion of studies reporting strong effect sizes over time. This result may be both contrary and comparable with previous findings. For example, in their study of 2,434 studies published across disciplines between 2000–2007, Fanelli [13] found that publications were more likely to report a positive result in social science disciplines such as psychology and behavioral science. Further, publications were more likely to report a positive result when the corresponding author was based in the USA. In an extension to this study, Fanelli [13] expanded the analysis to include studies published in the 1990s for a total number of 4,656. Over the reported period (1990–2007) there was a clear and significant increase in the proportion of publications reporting a significant result (70.2% - 88.6%). Here, investigators found a clear and significant higher average frequency of positive results when moving toward the social sciences (e.g., from physical to biological to social sciences) with 90% of the studies published within the social sciences during the final year of the analysis reporting a positive result. However, coupled with the overall finding of publication bias and an increase in publication bias over time, investigators found a marked difference in this proportion and rate across countries, with European countries and the EU as a region publishing fewer positive results [13] compared to other countries (e.g., Japan, USA) and regions (e.g., Asia). As all the publications included in this study are conducted in Sweden by Swedish investigators, the difference in finding of overall evidence of publication bias as well as change in positive publications over time may be explained by research culture in the social sciences in Sweden.

Finally, we were interested in the relationship between adherence to reporting standards and study findings as reporting standards have been advocated to reduce publication bias in the literature [e.g., 11, 20, 28, 29]. We found that adherence to study reporting standards has increased over time. Most notably in the areas of author provided conflict of interest statement, report of an a priori power calculation, report of primary outcome measures and report of study effect size. Despite these improvements, a large proportion of studies fail to report on a number of study characteristics as suggested by published reporting standards [e.g., 33, 39]. The average number of reported standards more than doubled during the investigated time-period. Few standards, in an exploratory sense, seem to be associated with study outcome. Most notably, studies reporting effect sizes tended to have significant and strong effects. If report of the standards will help to reduce publication bias, we would expect there to be no relationship or a negative relationship between reported standard and strength or significance of study finding. Another result is the lack of association between achieved study power and size of effect or significance. This implies that well-powered studies along with poor powered studies are being published despite their outcome which would indicate a lack of publication bias.

### Study limitations

Due the nature of this study, there are several limitations. First, this is an exploratory analysis of a population of effectiveness and efficacy research conducted in Sweden over the past 30 years. As such, this investigation includes a mix of different types of interventions, disciplines and organizations and publication bias may be unevenly distributed between the different categories of study included here. We have attempted to explore this heterogeneity through subgroup analyses which show that setting, prevention level and study design provide little explanatory power for the outcomes of significance, place of publication and time to publication.

Second, due to the retrospective nature of the study reported here there might be studies that we have missed that have not been published because of their results or for other reasons. Although we are confident that we have not missed many published studies, we lack knowledge of unpublished studies and grey literature was not included in the analyses. Investigations into publication bias in the social intervention literature should attempt to prospectively assess study bias or analyze studies as opposed to publications. It should be noted that our search for registered or funded studies during the search described above did not result in a finding of any additional unpublished studies.

Third, our study relies on information reported in the publications included here. This information is incomplete, but we are unable to further assess the extent of the incomplete reporting contained in the publications included in this analysis. We do not however have any reason to believe that there is a systematic non-reporting of information in the publications included here.

## Conclusions

We found no evidence of positive outcome bias, place of publication bias, or time-lag bias in a large population of social intervention research conducted in Sweden between 1990 and 2019. Importantly, overall adherence to reporting standards was found to be related to whether the study reported significant findings, place of publication, and time to publish. This coupled with the improvement in adherence to reporting standards over time provides initial exploratory evidence of the importance of adherence to reporting standards as a possible buffer against publication bias. This should however be weighed against the limitations of this study for drawing solid conclusions and should instead be understood as initial exploratory findings.

## Supporting information

S1 File Data extraction and coding instrument. (DOCX)

S2 File Items used in the rating of adherence to reporting standards. (DOCX)

S1 Data (XLSX)
