# Supplementary material for: Publication bias, time-lag bias, and place-of-publication bias in social intervention research: An exploratory study of 527 Swedish articles published between 1990–2019
Source: PLoS One. 2023 Feb 6;18(2):e0281110. doi: 10.1371/journal.pone.0281110 (PMC9901762; doi:10.1371/journal.pone.0281110)
Supplement: S1 File — (DOCX) [file pone.0281110.s001.docx]

| **Författare, år** (t ex Svensson et al, 2010) |  | | | | **Löpnr / Granskare** |
| --- | --- | --- | --- | --- | --- |
| 1. Namnet på den studerade insatsen (Experiment-/interventionsgrupp) |  | | | | |
| 2. Namnet eller innehållet på kontroll-/jämförelsegruppers insatser (t ex TAU, väntelista, namnet på annan specifik insats). *Använd den minst intervenerande för fortsatt data-extrahering* | 1  2 | | | | |
| 3. Vilken är målgruppen? *T ex vuxna med panikångest* |  | | | | |
| **Gemensamma för RCT och non-RCT** | **Ja** | **Nej** | **Ej tillämpligt** | **O-**  **säker** | **Anteckningar** |
| 4. Rapporteras urvalskriterier som använts (individer, kliniker, verksamheter)? Ska avse den nivå man randomiserade/fördelade på |  |  |  |  |  |
| 5. Beskrivs hur samplets storlek beräknades (avser före studiestart)? *Sök på power* |  |  |  |  |  |
| 6. Rapporterar man i en tabell demografiska och relevanta kliniska variabler vid baslinjen, separat för studiegrupperna? |  |  |  |  |  |
|  | | | | | |
| 7. Används termen “primary outcome(s)” för att definiera studiens viktigaste utfall? *Sök på primary.*  *Om de ej specificerats eller om det finns flera – använd det utfallsmått som nämns först i Metoddelen (ej mått för bakgrundsinfo etc),*  *Om det först nämnda måttet uppenbart är mindre viktigt i relation för interventionens mål välj ett annat mer relevant.* |  |  |  |  | Ange primärt utfallsmått: |
| 8. Rapporteras psykometriska egenskaper hos det instrument som använts för att mäta det primära utfallet?  *Lägstanivå för Ja: a) anger ex vis Cronbach alpha för det studerade samplet; b) ett generellt uttalande om reliabilitet etc, om de samtidigt hänvisar till en artikel där psykometrin studerats* |  |  |  |  |  |
|  | | | | | |
| 9. Används termen ”imputation” i rapporteringen? *Sök på Imput* |  |  |  |  |  |
| 10. Används termerna ”Intention-to-treat”, ”Intent-to-treat” eller förkortningen ”ITT”? *Sök på intent och ITT* |  |  |  |  |  |
| 1. Är det en efficacy (forskarledd verksamhet) eller effectiveness studie (ordinarie verksamhet) |  | | | | |
| 1. Handlar det om universell, selektiv eller indikerad prevention |  | | | | |
| 13. Var de som gjorde uppföljningsmätningen blinda för deltagarnas grupptillhörighet? *Här avses bedömare som t ex gör skattningar eller genomför intervjuer med deltagarna. Om deltagarna självrapporterar med formulär eller svarar via internet kodas ej tillämpligt.* *Sök blind* |  |  |  |  |  |
| 14. Finns det beskrivet i flow-chart eller i text hur många deltagare som fördelades till varje studiegrupp? *Om studien har randomiserat eller fördelat kluster till studiegrupperna (t ex skolor) krävs att både antal kluster och antal individer anges per grupp för Ja.* |  |  |  |  | N exp grupp  N kontrl.grupp 1  N kontrl.grupp 2 |
| 15. Finns det beskrivet hur många deltagare av deltagare som föll bort vid uppföljningsmätningarna i respektive studiegrupp? *Här avses bortfallet vid mätningarna, ej de som hoppade av insatsen.* |  |  |  |  | N expgrupp  N kontrgrupp 1  N kontrl.grupp 2 |
| 16. Rapporteras antalet individer per studiegrupp som inkluderades i analysen av det primära utfallet? |  |  |  |  | N exp grupp  N kontrl.grupp 1  N kontrl.grupp 2 |
| 17. Längsta uppföljningstid mätt från förmätning i månader? *Ange tiden som inkluderar samtliga grupper (tex före väntelistan får behandling)* |  | | | | |
| 18. Startår för datainsamling |  | | | | |

| **Gemensamma för RCT och non-RCT** | **Ja** | | **Nej** | | **Ej tillämpligt** | | **O-**  **säker** | **Anteckningar** |
| --- | --- | --- | --- | --- | --- | --- | --- | --- |
| 19. Rapporteras i vilken utsträckning deltagarna fick avsedd insats? *T ex om antalet ”treatment completers”, antal sessioner etc beskrivs* |  | |  | |  | |  |  |
| 20. Om statistisk baslinjeskillnad - rapporteras statistiska metoder för att justera interventionens effekt på det primära utfallsmåttet? |  | |  | |  | |  |  |
| 21. Rapporteras resultat från statistiska jämförelser av baslinjedata för de som var kvar i studien och de som föll bort, för samplet som helhet? Avser de som deltog resp. inte deltog i uppföljningsmätning. *Koda ej tillämpligt om bortfallet är max 10%* |  | |  | |  | |  |  |
| 22. Rapporteras resultat från statistiska jämförelser av baslinjedata för de som föll bort och de som var kvar, inom respektive studiegrupp? Avser de som deltog resp. inte deltog i uppföljningsmätningarna. *Koda ej tillämpligt om bortfallet är max 10%* |  | |  | |  | |  |  |
|  | | | | | | | | |
| 23. Finns beskrivande statistik i form av M och SD per grupp vid kontinuer-liga utfall, eller antal händelser och N per grupp vid dikotoma utfall)? |  | |  | |  | |  |  |
| 24. Rapporteras effektstorlek (Cohen´s d, OR, RR) för det primära utfallsmåttet?  *Om det rapporteras effektstorlekar från flera uppföljningsmätningar, utgå från den/de som avser den sista mätningen.* |  | |  | |  | |  | Typ av effektstorlek: |
| 25. Finns en statistiskt säkerställd effekt på det primära utfallsmåttet?  *Om resultat rapporteras från flera uppföljningsmätningar, utgå från den sista mätningen samt interaktionen grupp*tid (inte om huvudeffekt av tid)* |  | |  | |  | |  | Om ja - är effekten till experimentgruppens förmån: |
| 26. Rapporterar man att man undersökt om det förekommit oönskade utfall/ komplikationer? *Sök adverse* |  | |  | |  | |  |  |
| 27. Rapporteras om intressekonflikt |  | |  | |  | |  | Om ja - förelåg sådant: |
| 28. Rapporteras om finansiärer?  *Ex. myndigheter är Socialstyrelsen, Alkoholkommiteen, Folkhälsomyndigheten, Regeringen)* |  | |  | |  | |  | FORTE / FAS  VR  Formas  Vinova  Universitet  Myndigheter  Kommuner / landst.  Stiftelser  Övr forskningsråd |
| 29. Publicerat protokoll i förväg (t ex clinicaltrails.gov) |  | |  | |  | |  |  |
| 30. Har studien granskats i en etisk kommitté? |  | |  | |  | |  |  |
| 31. Supplementary material |  | |  | |  | |  |  |
| **Specifikt för RCT** | **Ja** | **Nej** | | **osäker** | | **Anteckningar** | | |
| 32. Rapporteras hur randomiseringssekvensen genererades (slumptalsgenerator, singlat slant, dragit lott etc)? |  |  | |  | |  | | |
| 33. Rapporteras vilken typ av randomisering som använts, dvs. simple randomization eller randomisering med någon typ av restriktion som blockning, stratifiering, minimization *Se fråga 8b CONSORT 2010* |  |  | |  | |  | | |
| 34. Rapporteras om det var omöjligt för de som fördelade personer eller kliniker att förutse vem som hamnade i vilken grupp (*t ex genom att man använde personer som var oberoende av projektet, eller i ordningsföljd numrerade, ogenomskinliga kuvert vid lottning)?* |  |  | |  | |  | | |
| **Specifikt för icke-RCT** | **Ja** | **Nej** | | **osäker** | | **Anteckningar** | | |
| 35. Rapporteras metoden som använts för att fördela deltagare till studiegrupperna? (*t ex självselektion till grupp, varannan person/klinik, fyller upp interventionsgruppen först sedan kontrollgruppen).* |  |  | |  | |  | | |
| 36. Rapporteras försök att minska risken för selektionsbias (pga icke-randomisering) genom exempelvis matchning? |  |  | |  | |  | | |

|  |  |
| --- | --- |
|  |  |
|  |  |
|  |  |
