# Supplementary material for: Publication bias, time-lag bias, and place-of-publication bias in social intervention research: An exploratory study of 527 Swedish articles published between 1990–2019
Source: PLoS One. 2023 Feb 6;18(2):e0281110. doi: 10.1371/journal.pone.0281110 (PMC9901762; doi:10.1371/journal.pone.0281110)
Supplement: S2 File — (DOCX) [file pone.0281110.s002.docx]

# S2. Interrater reliability in 40 randomly selected assessments of publication quality.

| Variable | Agreement (%) | Kappa (K) | Assessment |
| --- | --- | --- | --- |
| Study start year | 80 | 0,58 | Måttligt bra |
| University | 100 | 1 | Perfekt |
| Institution/Department | 100 | 1 | Perfekt |
| Type of comparison group (waitlist, etc) | 100 | 1 | Perfekt |
| Prevention level (indiated, etc) | 85 | 0,64 | Bra |
| Field | 83 | 0,68 | Bra |
| Published study protocol | 95 | 0,89 | Mycket bra |
| Conflict of interest statement | 100 | 1 | Mkt bra - perfekt |
| Ethics approval | 98 | 0,93 | Mycket bra |
| Descriptive statistics reported | 100 | 1 | Perfekt |
| Effect size reported | 93 | 0,85 | Mycket bra |
| Significant result | 100 | 1 | Perfekt |
| Reported harmful effects | 95 | 0,81 | Mycket bra |
| Reported a primary outcome variable | 98 | 0,95 | Mycket bra |
| Reported the psychometric properties of the measurement instruments used | 98 | 0,95 | Mycket bra |
| A priori power calculation | 98 | 0,95 | Mycket bra |
| Inclusion and exclusion criteria | 90 | 0,79 | Bra |
| Baseline characteristics reported | 93 | 0,82 | Mycket bra |
| Intention to treat (ITT) | 95 | 0,90 | Mycket bra |
| Assessments were blinded | 83 | 0,67 | Bra |
| Intervention implemented as intended | 90 | 0,79 | Bra |
| Controlled with randomization | 100 | 1 | Perfekt |
| RCT: distribution blinded^a^ | 81 | 0,63 | Bra |
| CT: attempts to minimize selection bias^b^ | 100 | 1 | Perfekt |

^a^*n*=32; ^b^*n*=8
